# Associations between sleep changes and multimorbidity patterns in middle-aged and older Chinese adults

**DOI:** 10.3389/fpubh.2025.1609345

**Published:** 2025-09-03

**Authors:** Shuting Yin, Yanfang Zhang, Litao Du, Jianfan Zhou, Dexu Chen

**Affiliations:** ^1^School of Physical Education, Shandong University, Jinan, Shandong, China; ^2^The Affiliated Hospital of Jining Medical University, Jining, Shandong, China

**Keywords:** decreased sleep duration, sleep trajectories, multimorbidity, multimorbidity patterns, middle-aged and older adults

## Abstract

**Objective:**

This study aimed to identify multimorbidity patterns among Chinese middle-aged and older adults and examine their associations with prior changes and long-term trajectories in sleep duration.

**Methods:**

Data from 9,747 participants in the 2011 and 2020 waves of the China Health and Retirement Longitudinal Study were analyzed. Sleep duration was self-reported in 2011 and 2020 while chronic conditions were self-reported in 2020. Latent class analysis identified multimorbidity patterns. Logistic and multinomial logistic regression was used to analyze associations of sleep duration changes and trajectories with multimorbidity and multimorbidity patterns.

**Results:**

Five multimorbidity patterns were identified: relatively healthy class (55.41%), metabolism class (23.22%), arthritis-digestive class (10.67%), respiratory class (5.42%), multi-system morbidity class (5.28%). Sleep duration decreases of ≥1.0 h were significantly associated with higher odds of overall multimorbidity and specific multimorbidity patterns, particularly metabolism, arthritis-digestive, respiratory, and multi-system morbidity classes (FDR-*p* < 0.05). Additionally, compared to the healthy-healthy trajectory, short-short, and healthy-short sleep trajectories were significantly associated with higher odds of multimorbidity, particularly in arthritis-digestive, respiratory, and multi-system morbidity classes, and long-short sleep trajectories were significantly associated with higher odds of multi-system morbidity classes (FDR-*p* < 0.05).

**Conclusion:**

Among Chinese middle-aged and older adults, prior decreases in sleep duration (≥1.0 h) and unfavorable sleep trajectories (short-short and healthy-short) were statistically associated with higher odds of overall multimorbidity and specific patterns, particularly those involving metabolic, arthritis-digestive, respiratory, and multi-system conditions.

## 1 Introduction

Chronic diseases such as cardiovascular diseases, cancer, chronic respiratory diseases, and diabetes are characterized by prolonged courses, complex etiologies (1), and ongoing challenges to both physiological and psychological health. These conditions significantly affect quality of life and present substantial obstacles. Currently, chronic diseases have become a major public health issue in China, impacting economic and social development. They threaten the health of Chinese citizens, accounting for around 70% of the total disease burden and contributing to 87% of national mortality rates (2).

Multimorbidity, the co-occurrence of two or more chronic conditions in an individual, is especially common among older adults and has become a growing global public health concern affecting millions worldwide (3). Individuals with multimorbidity often face compounded challenges, including functional decline, increased disability, poorer mental health, reduced quality of life, and accelerated biological aging (4). Traditionally, multimorbidity has been measured using simple disease counts or weighted indices (5). While these methods are useful for identifying patients requiring complex care, they offer limited insight for clinical guideline development. Specifically, they fail to differentiate between individuals with the same number of conditions but different disease types, and do not capture the underlying associations among diseases (5, 6). In reality, certain chronic diseases tend to cluster due to shared risk factors or pathophysiological mechanisms, a phenomenon known as multimorbidity patterns (7). Identifying these patterns helps refine disease classification, improve management strategies, reduce overall burden, and enhance health outcomes. Studies investigating multimorbidity patterns among middle-aged and older adults in China have reported inconsistent findings. For example, Zhou et al. identified four distinct patterns (6), while Zhang et al. reported five (8). Despite these efforts, there is still no consensus, and the epidemiological landscape of multimorbidity patterns in China remains unclear (9). Further research is needed to clarify these patterns and provide evidence-based guidance for effective prevention and management strategies.

Epidemiological studies have demonstrated that unhealthy lifestyle behaviors—such as smoking, excessive alcohol consumption, and sleep deprivation—significantly increase the risk of chronic diseases (10). Among these factors, sleep plays a vital role in maintaining various physiological functions. Insufficient sleep has been associated with a range of chronic conditions, including cardiovascular and cerebrovascular diseases, hypertension, cancer, diabetes, and depression, and is known to elevate mortality risk (11). While the link between sleep duration and individual chronic diseases is well established, its relationship with multimorbidity remains underexplored. A few prospective studies have suggested that nocturnal sleep duration may be associated with the prevalence of multimorbidity (12, 13). However, these studies primarily focused on the number of coexisting diseases, without considering how sleep duration might relate to specific patterns of multimorbidity. As people age, sleep patterns tend to change, often involving shorter total sleep duration and increased sleep fragmentation (14). In China, adults have experienced an average loss of 1.5 h of sleep over the past decade, according to the Annual Sleep Report of China 2022 (15). Despite these shifts, there is a lack of research examining whether changes in sleep duration or long-term sleep trajectories are associated not only with multimorbidity, but also with distinct multimorbidity patterns. Therefore, this study aims to (1) identify distinct multimorbidity patterns among middle-aged and older adults, and (2) explore the associations between prior changes in sleep duration and sleep trajectories with multimorbidity and multimorbidity patterns. These findings may help deepen understanding of how sleep behaviors are associated with specific chronic disease clusters, and may indicate disease clusters where future studies of prevention and intervention strategies are warranted.

## 2 Materials and methods

### 2.1 Design

**Figure 1:**
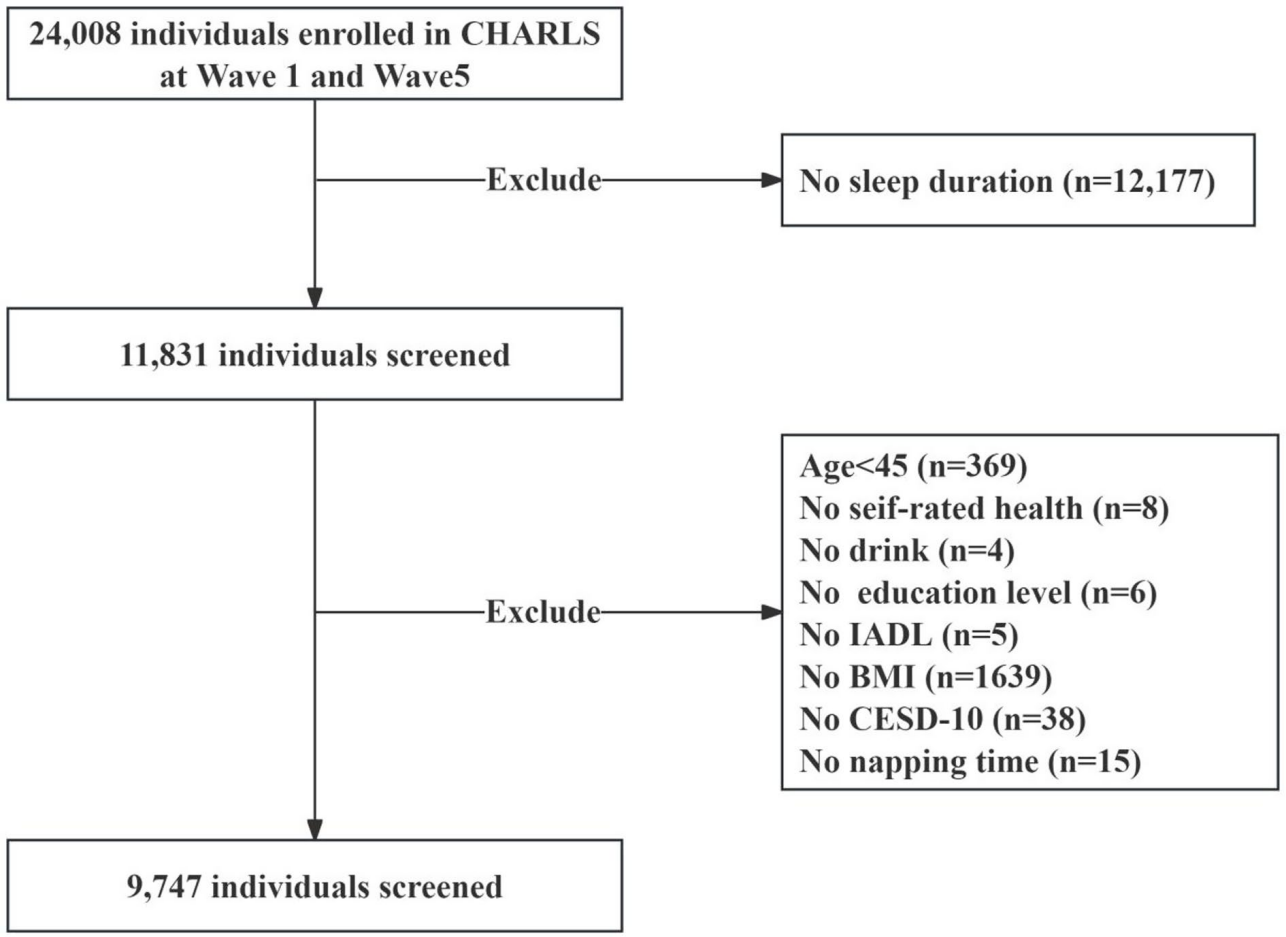
Flow chart of sample selection.

In this study, we used data from the China Health and Retirement Longitudinal Study (CHARLS), an ongoing cohort study covering community-dwelling individuals aged 45 years or older in 28 provinces in China. Since baseline (2011–2012), the survey collects information on various aspects of participants, including basic information, household information, health status and functioning, and personal income, every 2 years. Details of CHARLS recruitment and study design have been reported in other studies (11). For the current analysis, we utilized data from the baseline and the 2020 follow-up. Sleep duration was assessed at both baseline and in 2020, while information on chronic conditions was collected only in 2020. Therefore, this study adopts a cross-sectional design, with multimorbidity status determined at a single time point in 2020. Participants younger than 45 years of age who did not have sleep duration measured at baseline and 2020 and chronic disease data did were excluded, and ultimately, 9,747 participants were included in the final analyses (Figure 1). CHARLS was approved by the Ethical Review Board of Peking University (Approval No. IRB00001052-11,015), and all participants in the study signed an informed consent form.

### 2.2 Sleep duration and sleep trajectories

Self-reported nighttime sleep duration was obtained from the question “In the past month, how many h did you actually sleep at night (average number of h in a night)? Responses were recorded at both baseline (2011) and follow-up (2020). To assess changes in sleep duration over time, we calculated the difference by subtracting the 2020 nighttime sleep duration from the 2011 baseline value. Positive values indicated a decrease in sleep duration, while negative values indicated an increase. Based on the magnitude of change, participants were categorized into seven groups: increased ≥1.5 h, increased ≥1.0 and <1.5 h, increased ≥0.5 and <1.0 h, no change (absolute change <0.5), decreased ≥0.5 and <1.0 h, decreased ≥1.0 and <1.5 h, decreased ≥1.5 h (16). In addition, sleep duration was categorized as short (<6 h), healthy (≥6 & < 9 h), and long (≥9 h), following current recommendations (17, 18). We identified nine distinct sleep trajectories by tracking changes in sleep duration from baseline to follow-up over a 10-year period. These included: consistently short sleep (short-short), improvement from short to healthy (short-healthy), shift from short to long (short-long), decline from healthy to short (healthy-short), consistently healthy sleep (healthy-healthy), increase from healthy to long (healthy-long), consistently long sleep (long-long), improvement from long to healthy (long-healthy), and decline from long to short (long-short).

### 2.3 Multimorbidity and multimorbidity patterns

Chronic conditions were assessed based on self-reported physician diagnoses in 2020. The presence of a chronic condition was determined by the following question: “Have you been diagnosed by a doctor with any of the following conditions? Diseases surveyed in the questionnaire included: hypertension, dyslipidemia, diabetes/high blood sugar, cancer, chronic lung disease, liver disease (except fatty liver, tumors and cancers), heart disease (heart disease such as coronary artery disease, angina pectoris, heart failure, myocardial infarction, etc.), stroke, kidney disease, stomach/other digestive disorders, psychiatric problems, memory-related disorders, arthritis and asthma. Multimorbidity was defined as the presence of two or more chronic conditions. Latent class analysis (LCA) was applied to identify distinct multimorbidity patterns based on the reported conditions.

### 2.4 Covariates

Demographics included age (years), gender (male and female), education level (below primary, primary, junior high, higher and above), residence (urban and rural), marital (married and other), self-rated health (very good, good, fair, poor, very poor), body mass index (BMI), Center for Epidemiologic Studies Depression Scale (CESD-10), instrumental activities of daily living scale (IADL, whether or not you have difficulties), drink (yes, no), smoke (yes, no), and napping time (minutes). Napping time was obtained by asking “In the past month, how long (in minutes) have you napped after lunch?” All covariates were determined at baseline.

### 2.5 Statistical analysis

Continuous variables were presented as means ± standard deviations, while categorical variables were expressed as percentages. The Mann–Whitney U test and chi-square test were used to compare demographic characteristics between participants with and without multimorbidity. LCA was conducted to identify multimorbidity patterns based on chronic disease data collected in 2020. Models with one to six classes were tested, and model fit was assessed using the Bayesian Information Criterion (BIC), with lower BIC values indicating better fit. Based on BIC values and interpretability, a five-class model was selected to represent multimorbidity patterns (19). Logistic regression and multinomial logistic regression models were used to examine the associations of prior changes in sleep duration and long-term sleep trajectories with both overall multimorbidity and specific multimorbidity patterns. Two models were constructed: Model 1 adjusted for age and sex, and Model 2 additionally adjusted for residence, education level, marital status, self-rated health, smoking, alcohol consumption, napping frequency, BMI, CES-D10 score, and IADL. Subgroup analyses were conducted by stratifying participants into middle-aged (45–59 years) and older adults (≥60 years) to explore age-specific associations. To test the robustness of our findings, we conducted two sets of sensitivity analyses. First, we repeated the main analyses while adjusting for participants’ socioeconomic and health behavior factors at follow-up, including place of residence, smoke, and drink. Other covariates remained as measured at baseline. This approach allowed us to account for potential changes in lifestyle and living conditions over time. Second, we randomly divided the study sample into a training set (70%) and a testing set (30%). The primary analyses were then repeated separately within each subset to examine the consistency and reproducibility of the associations observed in the full sample. To account for multiple comparisons, we applied the False Discovery Rate (FDR) correction using the Benjamini-Hochberg procedure. Adjusted *p*-values were reported as FDR-adjusted *p*-values, and statistical significance was set at FDR-adjusted *p* < 0.05. All analyses were performed using Stata/MP 17.0 and R 4.2.2.

## 3 Results

### 3.1 Baseline characteristics of study

Of the 9,747 participants, 6,149 had multimorbidity. At baseline, the mean age was 57.87 years. Participants with multimorbidity were generally older, more likely to be female, had higher BMI, lived in urban areas, and had lower education levels. They also reported poorer self-rated health, worse IADL, poorer mental health, and were less likely to smoke or drink. Furthermore, they showed increased odds of prior decreased sleep duration. Prior unhealthy sleep trajectories were also more prevalent among those with multimorbidity, see Table 1 (all *p* < 0.05).

**Table 1:** Participant characteristics.

Characteristic	Total (9,747)	No-multimorbidity (3,598)	Multimorbidity (6,149)	*P*
Age (years)	57.87 ± 8.42	56.77 ± 8.56	58.51 ± 8.26	<0.01
Gender				<0.01
Male	4,495 (46.12)	1,780 (49.47)	2,715 (44.15)	
Female	5,252 (53.88)	1,818 (50.53)	3,434 (55.85)	
BMI (kg/m^2^)	23.58 ± 3.58	22.86 ± 3.20	24.00 ± 3.73	<0.01
Residence				0.02
Urban	3,340 (34.27)	1,181 (32.82)	2,159 (35.11)	
Rural	6,407 (65.73)	2,417 (67.18)	3,990 (64.89)	
Marital				0.17
Married	8,762 (89.89)	3,254 (90.44)	5,508 (89.58)	
Other	985 (10.11)	344 (9.56)	641 (10.42)	
Education level				0.02
Below primary	4,432 (45.47)	1,594 (44.30)	2,838 (46.15)	
Primary	2,135 (21.90)	761 (21.15)	1,374 (22.35)	
Junior high	2,106 (21.61)	824 (22.90)	1,282 (20.85)	
High and above	1,074 (11.02)	419 (11.65)	655 (10.65)	
Self-rated health				<0.01
Very good	611 (6.27)	353 (9.81)	258 (4.20)	
Good	1,690 (17.34)	860 (23.90)	830 (13.50)	
Fair	4,961 (50.90)	1,863 (51.78)	3,098 (50.38)	
Poor	2,128 (21.83)	469 (13.04)	1,659 (26.98)	
Very poor	357 (3.66)	53 (1.47)	304 (4.94)	
IADL	1,801 (18.48)	496 (13.79)	1,305 (21.22)	<0.01
CEDS-10	8.28 ± 6.21	6.83 ± 5.57	9.13 ± 6.41	<0.01
Drink	3,232 (33.16)	1,331 (36.99)	1,901 (30.92)	<0.01
Smoke	2,964 (30.41)	1,225 (34.05)	1,739 (28.28)	<0.01
Changes in sleep duration				<0.01
Increased ≥1.5 h	1,623 (16.65)	610 (16.95)	1,013 (16.47)	
Increased ≥1.0 and <1.5 h	1,346 (13.81)	528 (14.67)	818 (13.30)	
Increased ≥0.5 and <1.0 h	104 (1.07)	45 (1.25)	59 (0.96)	
No change	2,106 (21.61)	861 (23.93)	1,245 (20.25)	
Decreased ≥0.5 and <1.0 h	123 (1.26)	43 (1.20)	80 (1.30)	
Decreased ≥1.0 and <1.5 h	1,850 (18.98)	643 (17.87)	1,207 (19.63)	
Decreased ≥1.5 h	2,595 (26.62)	868 (24.12)	1,727 (28.09)	
Sleep trajectories				<0.01
Short-short	1,743 (17.88)	474 (13.17)	1,269 (20.64)	
Short-healthy	952 (9.77)	361 (10.03)	591 (9.61)	
Short-long	123 (1.26)	36 (1.00)	87 (1.41)	
Healthy-short	1,845 (18.93)	578 (16.06)	1,267 (20.60)	
Healthy-healthy	3,784 (38.82)	1,615 (44.89)	2,16 (35.27)	
Healthy-long	534 (5.48)	221 (6.14)	313 (5.09)	
Long-short	164 (1.68)	55 (1.53)	109 (1.77)	
Long-healthy	425 (4.36)	182 (5.06)	243 (3.95)	
Long-long	177 (1.82)	76 (2.11)	101 (1.64)	

Continuous variables are expressed as mean ± standard deviation and categorical variables as percentages.

### 3.2 Multimorbidity patterns identified by LCA

**Figure 2:**
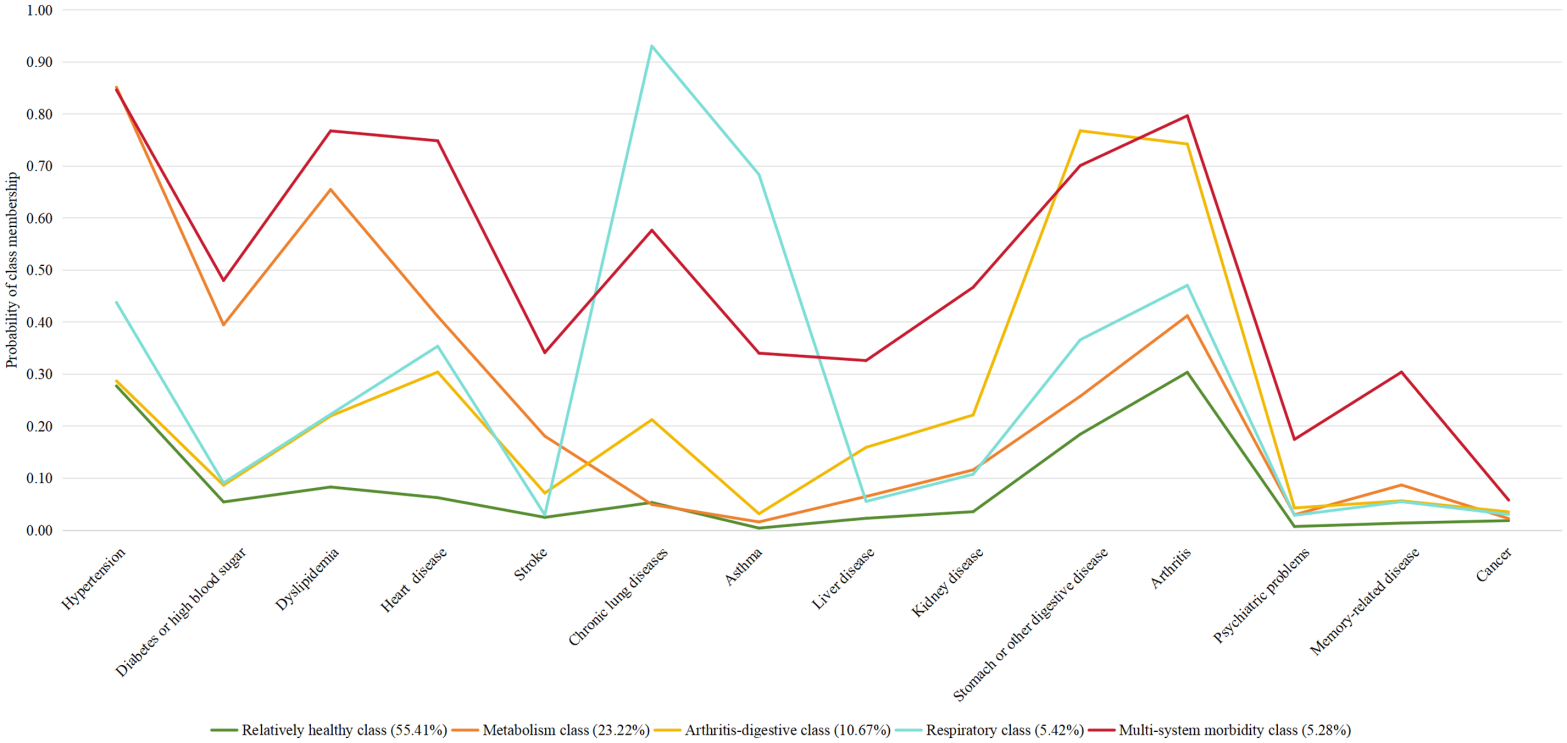
Five-class model of multimorbidity patterns.

Multimorbidity patterns was identified using LCA models ranging from one to six classes. The five-class model demonstrated the best fit based on the lowest BIC and yielded clinically interpretable results (Table 2). As shown in Figure 2, the classes were labeled according to multimorbidity patterns: Class 1, with low probabilities for all chronic diseases, was named the relatively healthy class; Class 2, featuring combinations of hypertension, diabetes, dyslipidemia, heart disease, or arthritis, was named the metabolism class; Class 3, with higher probabilities of gastric or digestive diseases and arthritis, was named the arthritis-digestive class; Class 4, with a higher probabilities of chronic lung disease and asthma alongside hypertension and arthritis, was named the respiratory class; Class 5, where all chronic diseases had higher probabilities, was named the multi-system morbidity class. Among all participants, 49.34% were classified as relatively healthy, 22.93% as metabolic, 16.33% as arthritis-digestive, 5.62% as respiratory, and 5.78% as multi-system morbidity (Figure 2).

**Table 2:** Comparison of fit statistics for latent class analysis models.

Classes	LogLik	AIC	BIC	Entropy	AvePP	Group membership
1	−54,997	110,021	110,122	–	–	100.00%
2	−52,608	105,275	105,483	0.63	86.30–90.50%	30.20–69.80%
3	−51,947	103,982	104,298	0.70	90.20–84.90–79.50%	65.36–12.95–21.69%
4	−51,615	103,346	103,770	0.71	76.00–74.90%–89.30–71.80%	19.14–8.00%–9.86–62.99%
5	−51,379	102,907	103,439	0.67	73.40–76.00%–80.90–83.60–68.30%	5.28–23.22%–10.67–5.42–55.41%
6	−51,329	102,833	103,473	0.63	81.00–72.20%–68.30–76.50%–83.00–55.90%	52.01–14.29%–5.03–15.43%–4.62–8.63%

Classes: Number of latent classes specified in the model; LogLik: Log-likelihood value of the model; higher (less negative) values indicate better model fit; AIC (Akaike Information Criterion): A model fit index; lower values suggest better fit, accounting for model complexity; BIC (Bayesian Information Criterion): A criterion for model selection; penalizes model complexity more strongly than AIC; Entropy: A measure of classification accuracy; values range from 0 to 1, with higher values indicating clearer class separation; AvePP (Average Posterior Probability): Average posterior probability of class membership; values >0.7 generally reflect good classification quality. Group membership: Proportion of participants assigned to each latent class, based on the highest posterior probability.

### 3.3 Association between prior changes in sleep duration and sleep trajectories with multimorbidity

Table 3 presents the associations between prior changes in sleep duration and sleep trajectories with multimorbidity. Compared to participants with no change in sleep duration, those with a decrease of ≥1.0 and <1.5 h (Model 1: OR = 1.29, 95% CI: 1.13–1.47; Model 2: OR = 1.30, 95% CI: 1.13–1.49) and those with a decrease of ≥1.5 h (Model 1: OR = 1.33, 95% CI: 1.18–1.50; Model 2: OR = 1.36, 95% CI: 1.20–1.54) were significantly associated with higher odds of multimorbidity. Similarly, compared to the healthy-healthy sleep trajectory, both the short-short (Model 1: OR = 1.83, 95% CI: 1.61–2.07; Model 2: OR = 1.43, 95% CI: 1.25–1.64) and healthy-short (Model 1: OR = 1.56, 95% CI: 1.39–1.76; Model 2: OR = 1.44, 95% CI: 1.27–1.63) trajectories were also significantly associated with higher odds of multimorbidity.

**Table 3:** Association between prior changes in sleep duration and sleep trajectories with multimorbidity.

Sleep-related variables	Model1		Model2	
	OR (95%CI)	FDR-P	OR (95%CI)	FDR-P
Changes in sleep duration				
No change	Ref		Ref	
Increased ≥1.5 h	1.05 (0.92, 1.21)	0.60	0.98 (0.85, 1.13)	0.92
Increased ≥1.0 and <1.5 h	1.05 (0.91, 1.21)	0.63	1.02 (0.88, 1.18)	0.87
Increased ≥0.5 and <1.0 h	0.90 (0.60, 1.34)	0.74	0.94 (0.61, 1.44)	0.90
Decreased ≥0.5 and <1.0 h	1.25 (0.85, 1.83)	0.43	1.21 (0.81, 1.80)	0.51
Decreased ≥1.0 and <1.5 h	1.29 (1.13, 1.47)	<0.01	1.30 (1.13, 1.49)	<0.01
Decreased ≥1.5 h	1.33 (1.18, 1.50)	<0.01	1.36 (1.20, 1.54)	<0.01
Sleep trajectories				
Healthy-healthy	Ref		Ref	
Short-short	1.83 (1.61, 2.07)	<0.01	1.43 (1.25, 1.64)	<0.01
Short-healthy	1.15 (0.99, 1.33)	0.15	0.98 (0.84, 1.15)	0.92
Short-long	1.50 (1.01, 2.23)	0.11	1.26 (0.83, 1.92)	0.45
Healthy-short	1.56 (1.39, 1.76)	<0.01	1.44 (1.27, 1.63)	<0.01
Healthy-long	0.96 (0.80, 1.16)	0.81	1.00 (0.82, 1.21)	1.00
Long-short	1.35 (0.97, 1.89)	0.17	1.38 (0.97, 1.96)	0.16
Long-healthy	0.99 (0.80, 1.21)	0.93	1.02 (0.83, 1.27)	0.90
Long-long	0.91 (0.67, 1.24)	0.69	0.95 (0.69, 1.31)	0.87

Model 1 adjusted for age and gender, Model 2 adjusted for age, gender, BMI, residence, education level, marital, smoke, drink, IADL, self-rated health, CESD-10, and napping time.

### 3.4 Association between prior changes in sleep duration and sleep trajectories with multimorbidity patterns

#### 3.4.1 Association between prior changes in sleep duration with multimorbidity patterns

Table 4 shows the associations between prior changes in sleep duration and patterns of multimorbidity. Compared to participants with no change in sleep duration, those who experienced a decrease of ≥1.0 and <1.5 h were significantly associated with higher odds of the metabolism class (Model 1: OR = 1.24, 95% CI: 1.06–1.45; Model 2: OR = 1.25, 95% CI: 1.07–1.47), arthritis-digestive class (Model 1: OR = 1.29, 95% CI: 1.05–1.59; Model 2: OR = 1.29, 95% CI: 1.04–1.59), and multi-system morbidity class (Model 1: OR = 1.45, 95% CI: 1.27–2.19; Model 2: OR = 1.70, 95% CI: 1.29–2.26). Similarly, a decrease in sleep duration of ≥1.5 h was significantly associated with higher odds of the metabolism class (Model 1: OR = 1.22, 95% CI: 1.06–1.41; Model 2: OR = 1.28, 95% CI: 1.10–1.49), respiratory class (Model 1: OR = 1.44, 95% CI: 1.10–1.88; Model 2: OR = 1.42, 95% CI: 1.08–1.86), and multi-system morbidity class (Model 1: OR = 1.67, 95% CI: 1.27–2.19; Model 2: OR = 1.70, 95% CI: 1.29–2.26).

**Table 4:** Association of prior changes in sleep duration with multimorbidity patterns.

Multimorbidity patterns (ref: relatively healthy class)	Changes in sleep duration	Model 1		Model 2	
		OR (95%CI)	FDR-P	OR (95%CI)	FDR-P
Metabolism class	No change	Ref		Ref	
	Increased ≥1.5 h	0.92 (0.78, 1.09)	0.47	0.96 (0.81, 1.14)	0.78
	Increased ≥1.0 and <1.5 h	0.98 (0.82, 1.16)	0.86	0.98 (0.81, 1.17)	0.86
	Increased ≥0.5 and <1.0 h	1.10 (0.68, 1.79)	0.78	1.22 (0.73, 2.03)	0.54
	Decreased ≥1.0 and <1.5 h	1.23 (0.79, 1.92)	0.49	1.31 (0.82, 2.08)	0.38
	Decreased ≥1.5 h	1.24 (1.06, 1.45)	0.02	1.25 (1.07, 1.47)	0.02
	Increased ≥1.5 h	1.22 (1.06, 1.41)	0.02	1.28 (1.10, 1.49)	<0.01
Arthritis-digestive class	No change	Ref		Ref	
	Increased ≥1.5 h	0.91 (0.73, 1.14)	0.54	0.79 (0.63, 0.99)	0.10
	Increased ≥1.0 and <1.5 h	0.95 (0.75, 1.20)	0.78	0.90 (0.71, 1.15)	0.55
	Increased ≥0.5 and <1.0 h	0.87 (0.43, 1.78)	0.80	0.85 (0.41, 1.78)	0.78
	Decreased ≥0.5 and <1.0 h	1.12 (0.60, 2.06)	0.81	1.01 (0.54, 1.90)	0.97
	Decreased ≥1.0 and <1.5 h	1.29 (1.05, 1.59)	0.03	1.29 (1.04, 1.59)	0.04
	Decreased ≥1.5 h	1.18 (0.97, 1.43)	0.19	1.18 (0.97, 1.44)	0.18
Respiratory class	No change	Ref		Ref	
	Increased ≥1.5 h	1.21 (0.90, 1.62)	0.34	1.07 (0.79, 1.44)	0.76
	Increased ≥1.0 and <1.5 h	1.25 (0.91, 1.70)	0.28	1.20 (0.88, 1.64)	0.38
	Increased ≥0.5 and <1.0 h	0.40 (0.10, 1.65)	0.33	0.42 (0.10, 1.77)	0.38
	Decreased ≥0.5 and <1.0 h	0.95 (0.37, 2.41)	0.92	0.90 (0.35, 2.32)	0.87
	Decreased ≥1.0 and <1.5 h	1.21 (0.90, 1.62)	0.35	1.20 (0.89, 1.62)	0.36
	Decreased ≥1.5 h	1.44 (1.10, 1.88)	0.02	1.42 (1.08, 1.86)	0.03
Multi-system morbidity class	No change	Ref		Ref	
	Increased ≥1.5 h	1.05 (0.77, 1.45)	0.83	0.88 (0.63, 1.23)	0.60
	Increased ≥1.0 and <1.5 h	1.11 (0.79, 1.55)	0.68	1.01 (0.71, 1.43)	0.95
	Increased ≥0.5 and <1.0 h	1.85 (0.86, 4.00)	0.22	1.79 (0.78, 4.07)	0.26
	Decreased ≥0.5 and <1.0 h	1.87 (0.90, 3.89)	0.18	1.95 (0.91, 4.18)	0.16
	Decreased ≥1.0 and <1.5 h	1.45 (1.07, 1.95)	0.04	1.43 (1.05, 1.95)	0.05
	Decreased ≥1.5 h	1.67 (1.27, 2.19)	<0.01	1.70 (1.29, 2.26)	<0.01

Model 1 adjusted for age and gender, Model 2 adjusted for age, gender, BMI, residence, education level, marital, smoke, drink, IADL, self-rated health, CESD-10, and napping time.

#### 3.4.2 Association between prior sleep trajectories with multimorbidity patterns

Table 5 presents the associations between prior sleep trajectories and multimorbidity patterns. Compared to the healthy-healthy sleep trajectory, the short-short trajectory was significantly associated with higher odds of arthritis-digestive class (Model 1: OR = 2.02, 95% CI: 1.69–2.43; Model 2: OR = 1.31, 95% CI: 1.07–1.60), respiratory class (Model 1: OR = 1.89, 95% CI: 1.47–2.44; Model 2: OR = 1.44, 95% CI: 1.10–1.89), multi-system morbidity class (Model 1: OR = 2.84, 95% CI: 2.20–3.66; Model 2: OR = 1.90, 95% CI: 1.44–2.50). The healthy-short trajectory was also significantly associated with higher odds of arthritis-digestive class (Model 1: OR = 1.67, 95% CI: 1.38–2.01; Model 2: OR = 1.46, 95% CI: 1.20–1.76), respiratory class (Model 1: OR = 1.69, 95% CI: 1.31–2.19; Model 2: OR = 1.54, 95% CI: 1.19–2.00), multi-system morbidity class (Model 1: OR = 2.54, 95% CI: 1.97–3.28; Model 2: OR = 2.21, 95% CI: 1.69–2.88). In addition, the long-short trajectory was significantly associated with higher odds of multi-system morbidity class (Model 1: OR = 2.76, 95% CI: 1.52–5.02; Model 2: OR = 2.86, 95% CI: 1.51–5.42).

**Table 5:** Association of prior sleep trajectories with multimorbidity patterns.

Multimorbidity patterns (ref: relatively healthy class)	Sleep trajectories	Model 1		Model 2	
		OR (95%CI)	FDR-P	OR (95%CI)	FDR-P
Metabolism class	Healthy-healthy	Ref		Ref	
	Short-short	1.09 (0.94, 1.26)	0.38	1.09 (0.93, 1.28)	0.39
	Short-healthy	0.95 (0.80, 1.14)	0.71	0.97 (0.81, 1.18)	0.84
	Short-long	0.98 (0.62, 1.54)	0.94	1.08 (0.67, 1.73)	0.83
	Healthy-short	1.23 (1.07, 1.41)	<0.01	1.24 (1.07, 1.43)	0.01
	Healthy-long	0.79 (0.63, 1.00)	0.10	0.86 (0.67, 1.09)	0.33
	Long-short	1.20 (0.82, 1.77)	0.48	1.44 (0.96, 2.17)	0.15
	Long-healthy	0.96 (0.75, 1.23)	0.84	1.02 (0.79, 1.32)	0.92
	Long-long	0.75 (0.51, 1.11)	0.27	0.84 (0.56, 1.26)	0.54
Arthritis-digestive class	Healthy-healthy	Ref		Ref	
	Short-short	2.02 (1.69, 2.43)	<0.01	1.31 (1.07, 1.60)	0.02
	Short-healthy	1.08 (0.84, 1.39)	0.68	0.82 (0.63, 1.06)	0.24
	Short-long	1.22 (0.65, 2.30)	0.67	0.86 (0.45, 1.64)	0.76
	Healthy-short	1.67 (1.38, 2.01)	<0.01	1.46 (1.20, 1.76)	<0.01
	Healthy-long	0.79 (0.56, 1.13)	0.32	0.79 (0.55, 1.13)	0.33
	Long-short	1.33 (0.78, 2.28)	0.43	1.26 (0.72, 2.18)	0.56
	Long-healthy	0.93 (0.64, 1.34)	0.78	0.95 (0.65, 1.38)	0.84
	Long-long	1.34 (0.84, 2.16)	0.36	1.32 (0.81, 2.14)	0.41
Respiratory class	Healthy-healthy	Ref		Ref	
	Short-short	1.89 (1.47, 2.44)	<0.01	1.44 (1.10, 1.89)	0.02
	Short-healthy	1.36 (0.99, 1.88)	0.13	1.12 (0.80, 1.56)	0.63
	Short-long	2.01 (1.03, 3.92)	0.09	1.56 (0.79, 3.08)	0.32
	Healthy-short	1.69 (1.31, 2.19)	<0.01	1.54 (1.19, 2.00)	<0.01
	Healthy-long	1.42 (0.98, 2.06)	0.14	1.40 (0.96, 2.04)	0.16
	Long-short	1.68 (0.85, 3.32)	0.25	1.63 (0.82, 3.25)	0.28
	Long-healthy	0.92 (0.55, 1.55)	0.84	0.92 (0.55, 1.55)	0.83
	Long-long	0.90 (0.43, 1.89)	0.84	0.88 (0.42, 1.86)	0.82
Multi-system morbidity class	Healthy-healthy	Ref		Ref	
	Short-short	2.84 (2.20, 3.66)	<0.01	1.90 (1.44, 2.50)	<0.01
	Short-healthy	1.52 (1.08, 2.14)	0.04	1.16 (0.81, 1.66)	0.55
	Short-long	2.03 (0.98, 4.21)	0.12	1.54 (0.71, 3.31)	0.40
	Healthy-short	2.54 (1.97, 3.28)	<0.01	2.21 (1.69, 2.88)	<0.01
	Healthy-long	0.71 (0.41, 1.24)	0.36	0.71 (0.40, 1.25)	0.37
	Long-short	2.76 (1.52, 5.02)	<0.01	2.86 (1.51, 5.42)	<0.01
	Long-healthy	0.74 (0.39, 1.39)	0.48	0.81 (0.42, 1.53)	0.63
	Long-long	0.44 (0.14, 1.39)	0.28	0.44 (0.13, 1.43)	0.30

Model 1 adjusted for age and gender, Model 2 adjusted for age, gender, BMI, residence, education level, marital, smoke, drink, IADL, self-rated health, CESD-10, and napping time.

### 3.5 Subgroup analysis

Subgroup analyses showed that, compared to participants with no change in sleep duration, those with a decrease of ≥1.0 h had significantly higher odds of multimorbidity in both middle-aged and older adults. Similarly, short-short and healthy-short sleep trajectories were associated with higher odds of multimorbidity across both age groups (FDR-adjusted *p* < 0.05; Supplementary Tables S1, S4). A ≥ 1.5-h decrease in sleep was further associated with higher odds of the metabolism class in middle-aged adults and the multi-system morbidity class in older adults (Supplementary Tables S2, S5). Regarding sleep trajectories, the short-short pattern was associated to higher odds of respiratory and multi-system morbidity classes in middle-aged adults, and to multi-system morbidity in older adults. The healthy-short trajectory was associated with metabolism, arthritis-digestive, and multi-system morbidity classes in middle-aged adults, and with arthritis-digestive class in older adults. The long-short trajectory was significantly associated with multi-system morbidity in older adults (FDR-adjusted *p* < 0.05; Supplementary Tables S3, S6).

### 3.6 Sensitivity analysis

Sensitivity analyses adjusting for follow-up socioeconomic and behavioral factors (residence, smoke, and drink), while retaining baseline covariates, showed generally consistent directions of associations with the main analysis (Supplementary Tables S7–S9). Furthermore, the main analyses were repeated separately in the training (70%) and testing (30%) datasets. The associations between changes in sleep duration and sleep trajectories with overall multimorbidity remained consistent with the main analysis in both the training and testing datasets. Although some associations between sleep duration/trajectories and specific multimorbidity patterns were not statistically significant in the training or testing subsets, the directions of associations were generally consistent with the main findings (Supplementary Tables S10–S12).

## 4 Discussion

In this study, we examined multimorbidity patterns among Chinese middle-aged and older adults, as well as the associations between prior changes in sleep duration and sleep trajectories with multimorbidity and specific multimorbidity patterns. Our findings showed that the metabolism class and arthritis-digestive class were the most common multimorbidity patterns in this population. Prior sleep duration decreases of ≥1.0 h were significantly associated with higher odds of overall multimorbidity and specific multimorbidity patterns, particularly metabolism, arthritis-digestive, respiratory, and multi-system morbidity classes. Compared to the healthy-healthy trajectory, short-short, and healthy-short prior sleep trajectories were significantly associated with higher odds of multimorbidity, particularly in arthritis-digestive, respiratory, and multi-system morbidity classes.

As the global burden of chronic disease multimorbidity rises, there is a need to transition from single-disease management to a multimorbidity management model. Identifying patterns of chronic disease multimorbidity is essential. Using LCA, we identified five multimorbidity patterns in Chinese middle-aged and older adults, namely, relatively healthy class (49.34%), metabolism class (22.93%), arthritis-digestive class (16.33%), respiratory class (5.62%), and multi-system morbidity class (5.78%). Zhang et al. also identified five multimorbidity patterns in Chinese older adults, namely relatively healthy class (49.8%), vascular class (24.7%), respiratory class (5.6%), stomach-arthritis class (14.5%) and multi-system morbidity class (5.4%) (8), this finding supports our study. However, in a study of half a million Chinese adults, patterns of multimorbidity were identified as cardiometabolic multimorbidity, respiratory multimorbidity, gastrointestinal and hepatorenal multimorbidity, and mental and arthritis multimorbidity (20). This study included a broader age range, whereas our study focused on adults aged 45 and older. Additionally, while that study used hierarchical cluster analysis, our study employed LCA. Although variations in chronic disease focus across studies have led to differing multimorbidity patterns (21–24), the metabolism class remains consistently robust (25), which is not surprising given the common etiology of such disorders, and our findings provide evidence in support of this. Moreover, the respiratory class has been validated in countries such as Finland and Russia (25, 26), particularly among the older adults, where asthma can increase the risk of chronic obstructive pulmonary disease (COPD) (27), often overlapping to form asthma-COPD overlap syndrome (28). Our study also found a common combination of gastric or other digestive disorders with arthritis, which may be explained by the high prevalence of arthritis and digestive disorders in China (29, 30), and/or the gastrointestinal side effects of non-steroidal anti-inflammatory drugs commonly prescribed for arthritis (31). Finally, metabolism class and arthritis-digestive class are more common multimorbidity patterns among Chinese middle-aged and older adults, and healthcare professionals should focus on identifying and managing them.

Our study explored associations between prior decreased sleep duration and previously unexamined multimorbidity and multimorbidity patterns. It is well established that decreased sleep duration is common with age (32), possibly due to a decrease in the number of hypothalamic ventral preoptic nuclei, which may contribute to a decrease in sleep duration (33). Previous research indicates that a 1 h decrease in sleep duration is associated with a higher risk of adverse health outcomes such as cognitive impairment (34), metabolic syndrome (35), and non-alcoholic fatty liver disease (32). Our study found that prior sleep duration decreases of ≥1.0 h were significantly associated with higher odds of overall multimorbidity and specific multimorbidity patterns, particularly metabolism, arthritis-digestive, respiratory, and multi-system morbidity classes. These findings suggest that decreased sleep duration is associated with adverse multimorbidity patterns. Experimental research shows that after a week of partial sleep restriction (e.g., 4 h of sleep per night), a decrease in endothelium-dependent vasodilation is associated with strong activation of inflammatory and metabolic pathways, rather than autonomic status (36, 37). Sleep deprivation has also been shown to impact various biological pathways, including cardiovascular autonomic control, oxidative stress, inflammatory responses, and endothelial function, and is associated with cardiovascular and metabolic disorders (38). Persistent sleep reduction can activate inflammatory signaling pathways (39), and chronic shortening of sleep duration may increase sympathetic nervous system activity, raise nocturnal cortisol levels, and reduce cerebral glucose utilization, leading to insulin resistance (40). Changes in inflammatory responses, metabolic systems, and oxidative stress are associated with chronic disease development, making the association between decreased sleep duration and comorbid conditions plausible.

In addition, we constructed 10-year sleep trajectories to further explore the potential associations between different prior sleep patterns and multimorbidity and specific multimorbidity patterns. The results showed that, compared to the healthy-healthy trajectory, short-short, and healthy-short sleep trajectories were significantly associated with higher odds of multimorbidity and specific multimorbidity patterns. Specifically, both the short-short and healthy-short trajectories were significantly associated with higher odds of the arthritis-digestive class, the respiratory class, and the multi-system morbidity class. Moreover, the healthy-short trajectory was additionally associated with higher odds of the metabolism class, while the long-short trajectory also showed higher odds of the multi-system morbidity class. These findings are consistent with previous research. For example, a prospective study conducted among Chinese adults found that both healthy-short and short-short sleep trajectories were significantly associated with increased risks of cardiovascular disease events and all-cause mortality (41). Additionally, a 5-year longitudinal study further confirmed the association between the healthy-short trajectory and an elevated risk of all-cause mortality (42). Similar findings were observed in a study of middle-aged and older adults in the United States, where Xiao et al. reported that both healthy-short and short-short trajectories were significantly associated with an increased risk of developing diabetes, compared to the healthy-healthy group (43). Furthermore, another study based on a Chinese population indicated that consistently sleeping less than 6 h per day was associated with a higher risk of various chronic conditions—particularly mental disorders, digestive diseases, and arthritis (44). Together, these lines of evidence suggest that persistent short sleep duration may be associated with higher odds of multiple chronic diseases. Therefore, maintaining a stable and sufficient sleep pattern over time, especially avoiding transitions toward short sleep duration, may be associated with lower multimorbidity odds. Future studies are warranted to explore the underlying mechanisms of sleep trajectory changes and their potential role in multimorbidity development, which may help inform more targeted and effective public health strategies.

Our study has several strengths. First, we employed both sleep change and sleep trajectory analyses to capture complementary aspects of long-term sleep behavior—sleep change reflects the direction and magnitude of change, while trajectories represent distinct patterns across time. This dual approach offers a more comprehensive understanding of how sleep dynamics relate to multimorbidity. Second, we used LCA to identify distinct multimorbidity patterns, providing a person-centered perspective on chronic disease clustering and offering clinically meaningful insights into multimorbidity profiles. However, several limitations should be acknowledged. First, this was a cross-sectional study, which limits the ability to assess temporal or causal relationships between changes in sleep duration, sleep trajectories, and multimorbidity or its patterns. Second, the identification of chronic conditions relied on self-reported physician diagnoses, which may be subject to misclassification bias. Third, the analysis was restricted to a subset of chronic diseases, excluding conditions such as osteoporosis and cataracts; further research is needed to confirm the findings across a broader disease spectrum. Fourth, categorizing sleep trajectories into fixed types may oversimplify longitudinal variation. Future studies using latent growth or trajectory mixture modeling could better reflect heterogeneity in sleep pattern changes. Finally, all covariates were measured at baseline in 2011, whereas multimorbidity patterns were assessed in 2020, resulting in a time gap of approximately nine years. This temporal mismatch may have introduced bias due to changes in participants’ characteristics over time. To partially address this issue, we conducted a sensitivity analysis focusing on socioeconomic factors and health behaviors. Despite these limitations, the findings may have practical relevance. Regular monitoring of multimorbidity among older adults may help inform risk assessment, as the presence of one condition often coincides with others. Identifying individuals with hypertension who are also at elevated risk for diabetes or dyslipidemia may support early preventive efforts. Additionally, reduced sleep duration is a public health concern that warrants attention. Programs promoting healthy sleep habits could be considered as part of broader efforts to address multimorbidity.

## 5 Conclusion

The multimorbidity patterns among Chinese middle-aged and older adults can be categorized into five main classes: relatively healthy class, metabolism class, arthritis-digestive class, respiratory class, multi-system morbidity class. Prior sleep duration decreases of ≥1.0 h and unfavorable sleep trajectories—including short-short, and healthy-short were significantly associated with higher odds of overall multimorbidity and specific patterns, particularly those involving metabolism, arthritis-digestive, respiratory, and multi-system morbidity.

## Data Availability

The original contributions presented in the study are included in the article/Supplementary material, further inquiries can be directed to the corresponding author.
