# Supplementary material for: Associations between sleep changes and multimorbidity patterns in middle-aged and older Chinese adults
Source: Front Public Health. 2025 Sep 3;13:1609345. doi: 10.3389/fpubh.2025.1609345 (PMC12440773; doi:10.3389/fpubh.2025.1609345)
Supplement: Supplementary file 1 [file Table_1.docx]

**Table S1 Association between prior changes in sleep duration and sleep trajectories with multimorbidity in middle-aged adults.**

|  | Model1 | | Model2 | |
| --- | --- | --- | --- | --- |
|  | OR(95%CI) | FDR P | OR(95%CI) | FDR P |
| Changes in sleep duration |  |  |  |  |
| no change | Ref | | Ref | |
| increased ≥1.5 h | 1.15(0.96,1.37) | 0.28 | 1.00(0.83,1.21) | 0.95 |
| increased ≥1.0 and <1.5 h | 1.08(0.90,1.29) | 0.62 | 0.99(0.82,1.19) | 0.95 |
| increased ≥0.5 and <1.0 h | 1.08(0.64,1.80) | 0.94 | 1.23(0.72,2.11) | 0.63 |
| decreased ≥0.5 and <1.0 h | 1.21(0.74,1.98) | 0.66 | 1.20(0.72,2.00) | 0.68 |
| decreased ≥1.0 and <1.5 h | 1.28(1.08,1.49) | 0.01 | 1.26(1.07,1.49) | 0.02 |
| decreased ≥1.5 h | 1.41(1.21,1.64) | <0.01 | 1.40(1.19,1.64) | <0.01 |
| Sleep trajectories |  |  |  |  |
| Healthy-healthy | Ref | | Ref | |
| Short-short | 1.94(1.65,2.28) | <0.01 | 1.44(1.21,1.72) | <0.01 |
| Short-healthy | 1.16(0.96,1.40) | 0.26 | 0.94(0.77,1.14) | 0.76 |
| Short-long | 2.13(1.08,4.17) | 0.09 | 1.84(0.89,3.77) | 0.21 |
| Healthy-short | 1.62(1.40,1.88) | <0.01 | 1.48(1.27,1.72) | <0.01 |
| Healthy-long | 1.02(0.79,1.32) | 0.95 | 1.03(0.79,1.35) | 0.95 |
| Long-short | 1.56(1.02,2.40) | 0.12 | 1.57(1.00,2.48) | 0.13 |
| Long-healthy | 0.93(0.73,1.19) | 0.78 | 0.93(0.72,1.20) | 0.80 |
| Long-long | 1.05(0.69,1.59) | 0.95 | 1.07(0.69,1.65) | 0.92 |

Note: Model 1 adjusted for age and gender, Model 2 adjusted for age, gender, BMI, residence, education level, marital, smoke, drink, IADL, self-rated health, CESD-10, and napping time.

**Table S2 Association of prior changes in sleep duration with multimorbidity in middle-aged adults adults.**

| Multimorbidity patterns  (ref: relatively healthy class) | Changes in sleep duration | Model1 | | Model2 | |
| --- | --- | --- | --- | --- | --- |
|  |  | OR(95%CI) | FDR P | OR(95%CI) | FDR P |
| Metabolism class | no change | Ref | | Ref | |
|  | increased ≥1.5 h | 1.05(0.84,1.31) | 0.87 | 1.02(0.80,1.30) | 0.95 |
|  | increased ≥1.0 and <1.5 h | 0.95(0.75,1.19) | 0.82 | 0.87(0.68,1.11) | 0.49 |
|  | increased ≥0.5 and <1.0 h | 1.51(0.83,2.72) | 0.37 | 1.82(0.98,3.39) | 0.15 |
|  | decreased ≥0.5 and <1.0 h | 0.89(0.46,1.71) | 0.90 | 1.01(0.51,1.99) | 0.97 |
|  | decreased ≥1.0 and <1.5 h | 1.27(1.04,1.55) | 0.06 | 1.26(1.03,1.56) | 0.08 |
|  | decreased ≥1.5 h | 1.34(1.16,1.62) | <0.01 | 1.36(1.12,1.66) | <0.01 |
| Arthritis-digestive class | no change | Ref | | Ref | |
|  | increased ≥1.5 h | 1.02(0.76,1.37) | 0.95 | 0.84(0.62,1.14) | 0.49 |
|  | increased ≥1.0 and <1.5 h | 0.96(0.71,1.30) | 0.95 | 0.86(0.63,1.17) | 0.56 |
|  | increased ≥0.5 and <1.0 h | 1.03(0.43,2.50) | 0.97 | 1.08(0.44,2.67) | 0.95 |
|  | decreased ≥0.5 and <1.0 h | 1.00(0.44,2.26) | 1.00 | 1.01(0.54,1.90) | 0.95 |
|  | decreased ≥1.0 and <1.5 h | 1.28(0.99,1.65) | 0.16 | 1.29(1.04,1.59) | 0.22 |
|  | decreased ≥1.5 h | 1.16(0.91,1.48) | 0.46 | 1.18(0.97,1.44) | 0.58 |
| Respiratory class | no change | Ref | | Ref | |
|  | increased ≥1.5 h | 1.63(1.06,2.51) | 0.08 | 1.31(0.85,2.04) | 0.42 |
|  | increased ≥1.0 and <1.5 h | 1.64(1.07,2.52) | 0.07 | 1.46(0.95,2.25) | 0.21 |
|  | increased ≥0.5 and <1.0 h | 0.40(0.10,1.65) | 0.97 | 0.42(0.10,1.77) | 0.98 |
|  | decreased ≥0.5 and <1.0 h | 0.42(0.06,3.10) | 0.61 | 0.40(0.05,3.01) | 0.58 |
|  | decreased ≥1.0 and <1.5 h | 1.02(0.66,1.60) | 0.97 | 1.01(0.64,1.58) | 0.97 |
|  | decreased ≥1.5 h | 1.63(1.11,2.38) | 0.04 | 1.55(1.05,2.28) | 0.08 |
| Multi-system morbidity class | no change | Ref | | Ref | |
|  | increased ≥1.5 h | 0.98(0.61,1.56) | 0.97 | 0.71(0.43,1.16) | 0.38 |
|  | increased ≥1.0 and <1.5 h | 1.04(0.66,1.64) | 0.95 | 0.85(0.53,1.37) | 0.68 |
|  | increased ≥0.5 and <1.0 h | 0.91(0.21,3.89) | 0.95 | 1.15(0.26,5.14) | 0.95 |
|  | decreased ≥0.5 and <1.0 h | 1.89(0.72,4.99) | 0.40 | 2.24(0.82,6.11) | 0.26 |
|  | decreased ≥1.0 and <1.5 h | 1.26(0.85,1.88) | 0.48 | 1.22(0.80,1.85) | 0.55 |
|  | decreased ≥1.5 h | 1.52(1.06,2.18) | 0.08 | 1.51(1.03,2.20) | 0.10 |

Note: Model 1 adjusted for age and gender, Model 2 adjusted for age, gender, BMI, residence, education level, marital, smoke, drink, IADL, self-rated health, CESD-10, and napping time.

**Table S3 Association of prior sleep trajectories with multimorbidity patterns in middle-aged adults.**

| Multimorbidity patterns  (ref: relatively healthy class) | Sleep trajectories | Model1 | | Model2 | |
| --- | --- | --- | --- | --- | --- |
|  |  | OR（95%CI） | FDR P | OR（95%CI） | FDR P |
| Metabolism class | Healthy-healthy | Ref | | Ref | |
|  | Short-short | 1.14(0.94,1.39) | 0.40 | 1.06(0.86,1.32) | 0.79 |
|  | Short-healthy | 1.01(0.80,1.28) | 0.97 | 0.95(0.74,1.23) | 0.87 |
|  | Short-long | 1.10(0.51,2.37) | 0.95 | 1.12(0.49,2.56) | 0.94 |
|  | Healthy-short | 1.43(1.20,1.70) | <0.01 | 1.40(1.17,1.68) | <0.01 |
|  | Healthy-long | 0.83(0.59,1.18) | 0.53 | 0.89(0.62,1.28) | 0.73 |
|  | Long-short | 1.31(0.79,2.19) | 0.52 | 1.57(0.91,2.71) | 0.21 |
|  | Long-healthy | 0.84(0.61,1.16) | 0.52 | 0.83(0.59,1.16) | 0.50 |
|  | Long-long | 0.77(0.44,1.35) | 0.58 | 0.81(0.45,1.47) | 0.68 |
| Arthritis-digestive class | Healthy-healthy | Ref | | Ref | |
|  | Short-short | 2.00(1.57,2.54) | <0.01 | 1.28(0.99,1.66) | 0.15 |
|  | Short-healthy | 1.08(0.78,1.49) | 0.84 | 0.79(0.56,1.10) | 0.36 |
|  | Short-long | 1.43(0.54,3.77) | 0.68 | 0.96(0.35,2.64) | 0.97 |
|  | Healthy-short | 1.61(1.28,2.03) | <0.01 | 1.42(1.12,1.81) | 0.02 |
|  | Healthy-long | 1.05(0.67,1.65) | 0.95 | 1.04(0.66,1.66) | 0.95 |
|  | Long-short | 1.88(1.02,3.46) | 0.12 | 1.72(0.91,3.24) | 0.21 |
|  | Long-healthy | 0.90(0.57,1.40) | 0.82 | 0.88(0.56,1.39) | 0.78 |
|  | Long-long | 1.46(0.79,2.70) | 0.44 | 1.39(0.74,2.62) | 0.54 |
| Respiratory class | Healthy-healthy | Ref | | Ref | |
|  | Short-short | 2.48(1.74,3.54) | <0.01 | 1.71(1.17,2.49) | 0.02 |
|  | Short-healthy | 1.80(1.16,2.78) | 0.03 | 1.32(0.85,2.07) | 0.42 |
|  | Short-long | 0.81(0.11,6.04) | 0.95 | 0.53(0.07,4.02) | 0.76 |
|  | Healthy-short | 1.75(1.22,2.52) | 0.01 | 1.55(1.07,2.25) | 0.06 |
|  | Healthy-long | 2.06(1.19,3.56) | 0.04 | 1.99(1.14,3.48) | 0.05 |
|  | Long-short | 1.28(0.39,4.19) | 0.87 | 1.15(0.35,3.82) | 0.95 |
|  | Long-healthy | 1.05(0.53,2.06) | 0.95 | 1.00(0.51,1.98) | 1.00 |
|  | Long-long | 0.32(0.04,2.35) | 0.49 | 0.29(0.04,2.16) | 0.45 |
| Multi-system morbidity class | Healthy-healthy | Ref | | Ref | |
|  | Short-short | 2.99(2.12,4.21) | <0.01 | 1.65(1.13,2.42) | 0.03 |
|  | Short-healthy | 1.04(0.61,1.79) | 0.95 | 0.70(0.40,1.22) | 0.40 |
|  | Short-long | 1.68(0.39,7.22) | 0.69 | 0.80(0.17,3.88) | 0.95 |
|  | Healthy-short | 2.47(1.76,3.46) | <0.01 | 2.01(1.41,2.86) | <0.01 |
|  | Healthy-long | 1.03(0.49,2.16) | 0.97 | 1.02(0.47,2.19) | 0.97 |
|  | Long-short | 2.43(1.01,5.85) | 0.13 | 2.44(0.97,6.17) | 0.14 |
|  | Long-healthy | 0.88(0.42,1.85) | 0.90 | 0.90(0.42,1.94) | 0.94 |
|  | Long-long | 0.33(0.05,2.43) | 0.50 | 0.32(0.04,2.38) | 0.49 |

Note: Model 1 adjusted for age and gender, Model 2 adjusted for age, gender, BMI, residence, education level, marital, smoke, drink, IADL, self-rated health, CESD-10, and napping time.

**Table S4 Association between prior changes in sleep duration and sleep trajectories with multimorbidity in older adults.**

|  | Model1 | | Model2 | |
| --- | --- | --- | --- | --- |
|  | OR(95%CI) | FDR P | OR(95%CI) | FDR P |
| Changes in sleep duration |  |  |  |  |
| no change | Ref | | Ref | |
| increased ≥1.5 h | 1.00(0.81,1.24) | 0.99 | 1.05(0.84,1.21) | 0.77 |
| increased ≥1.0 and <1.5 h | 1.04(0.83,1.32) | 0.80 | 1.15(0.90,1.47) | 0.44 |
| increased ≥0.5 and <1.0 h | 0.70(0.37,1.31) | 0.44 | 0.65(0.33,1.29) | 0.39 |
| decreased ≥0.5 and <1.0 h | 1.29(0.70,2.38) | 0.58 | 1.18(0.62,2.25) | 0.75 |
| decreased ≥1.0 and <1.5 h | 1.39(1.10,1.74) | 0.02 | 1.44(1.13,1.82) | 0.01 |
| decreased ≥1.5 h | 1.26(1.02,1.54) | 0.08 | 1.37(1.11,1.70) | 0.01 |
| Sleep trajectories |  |  |  |  |
| Healthy-healthy | Ref | | Ref | |
| Short-short | 1.72(1.41,2.09) | <0.01 | 1.49(1.20,1.85) | <0.01 |
| Short-healthy | 1.16(0.92,1.48) | 0.38 | 1.10(0.85,1.43) | 0.61 |
| Short-long | 1.27(0.77,2.07) | 0.54 | 1.19(0.71,2.00) | 0.64 |
| Healthy-short | 1.49(1.22,1.83) | <0.01 | 1.42(1.14,1.76) | <0.01 |
| Healthy-long | 0.93(0.71,1.22) | 0.74 | 1.04(0.78,1.38) | 0.86 |
| Long-short | 1.15(0.69,1.94) | 0.72 | 1.22(0.70,2.11) | 0.62 |
| Long-healthy | 1.14(0.78,1.66) | 0.64 | 1.33(0.90,1.97) | 0.31 |
| Long-long | 0.81(0.51,1.27) | 0.55 | 0.92(0.57,1.49) | 0.81 |

Note: Model 1 adjusted for age and gender, Model 2 adjusted for age, gender, BMI, residence, education level, marital, smoke, drink, IADL, self-rated health, CESD-10, and napping time.

**Table S5 Association of prior changes in sleep duration with multimorbidity patterns in older adults.**

| Multimorbidity patterns  (ref: relatively healthy class) | Changes in sleep duration | Model1 | | Model2 | |
| --- | --- | --- | --- | --- | --- |
|  |  | OR(95%CI) | FDR P | OR(95%CI) | FDR P |
| Metabolism class | no change | Ref | | Ref | |
|  | increased ≥1.5 h | 0.84(0.66,1.07) | 0.32 | 0.97(0.75,1.26) | 0.89 |
|  | increased ≥1.0 and <1.5 h | 1.05(0.81,1.36) | 0.81 | 1.19(0.90,1.56) | 0.39 |
|  | increased ≥0.5 and <1.0 h | 0.70(0.31,1.59) | 0.57 | 0.72(0.30,1.71) | 0.62 |
|  | decreased ≥0.5 and <1.0 h | 1.69(0.89,3.22) | 0.24 | 1.64(0.84,3.22) | 0.31 |
|  | decreased ≥1.0 and <1.5 h | 1.24(0.97,1.60) | 0.20 | 1.29(0.99,1.68) | 0.14 |
|  | decreased ≥1.5 h | 0.80(0.57,1.12) | 0.57 | 1.24(0.97,1.58) | 0.19 |
| Arthritis-digestive class | no change | Ref | | Ref | |
|  | increased ≥1.5 h | 0.94(0.65,1.38) | 0.37 | 0.75(0.53,1.07) | 0.25 |
|  | increased ≥1.0 and <1.5 h | 0.96(0.71,1.30) | 0..84 | 0.99(0.67,1.46) | 0.98 |
|  | increased ≥0.5 and <1.0 h | 0.65(0.19,2.22) | 0.64 | 0.57(0.16,2.00) | 0.56 |
|  | decreased ≥0.5 and <1.0 h | 1.34(0.53,3.44) | 0.68 | 1.24(0.47,3.25) | 0.76 |
|  | decreased ≥1.0 and <1.5 h | 1.36(0.97,1.91) | 0.19 | 1.39(0.98,1.98) | 0.15 |
|  | decreased ≥1.5 h | 1.22(0.89,1.67) | 0.39 | 1.30(0.94,1.79) | 0.25 |
| Respiratory class | no change | Ref | | Ref | |
|  | increased ≥1.5 h | 1.02(0.68,1.52) | 0.95 | 0.98(0.65,1.47) | 0.98 |
|  | increased ≥1.0 and <1.5 h | 0.97(0.62,2.53) | 0.94 | 1.02(0.64,1.62) | 0.94 |
|  | increased ≥0.5 and <1.0 h | 0.65(0.15,2.83) | 0.71 | 0.62(0.14,2.76) | 0.72 |
|  | decreased ≥0.5 and <1.0 h | 1.53(0.50,4.63) | 0.62 | 1.45(0.47,4.44) | 0.71 |
|  | decreased ≥1.0 and <1.5 h | 1.48(0.98,2.22) | 0.15 | 1.50(0.99,2.27) | 0.14 |
|  | decreased ≥1.5 h | 1.33(0.91,1.95) | 0.29 | 1.38(0.94,2.02) | 0.22 |
| Multi-system morbidity class | no change | Ref | | Ref | |
|  | increased ≥1.5 h | 1.20(0.77,1.88) | 0.58 | 1.19(0.74,1.89) | 0.62 |
|  | increased ≥1.0 and <1.5 h | 1.24(0.75,2.04) | 0.57 | 1.37(0.81,2.29) | 0.37 |
|  | increased ≥0.5 and <1.0 h | 2.86(1.10,7.47) | 0.09 | 2.28(0.79,6.54) | 0.22 |
|  | decreased ≥0.5 and <1.0 h | 1.99(0.65,6.11) | 0.39 | 1.79(0.56,5.74) | 0.49 |
|  | decreased ≥1.0 and <1.5 h | 1.78(0.14,2.80) | 0.04 | 1.87(1.17,2.99) | 0.03 |
|  | decreased ≥1.5 h | 1.94(1.29,2.92) | <0.01 | 2.19(1.43,3.36) | <0.01 |

Note: Model 1 adjusted for age and gender, Model 2 adjusted for age, gender, BMI, residence, education level, marital, smoke, drink, IADL, self-rated health, CESD-10, and napping time.

**Table S6 Association of prior sleep trajectories with multimorbidity patterns in older adults.**

| Multimorbidity patterns  (ref: relatively healthy class) | Sleep trajectories | Model1 | | Model2 | |
| --- | --- | --- | --- | --- | --- |
|  |  | OR（95%CI） | FDR P | OR（95%CI） | FDR P |
| Metabolism class | Healthy-healthy | Ref | | Ref | |
|  | Short-short | 1.03(0.83,1.28) | 0.86 | 1.15(0.91,1.47) | 0.41 |
|  | Short-healthy | 0.90(0.69,1.19) | 0.62 | 1.03(0.77,1.38) | 0.89 |
|  | Short-long | 0.93(0.53,1.63) | 0.86 | 1.19(0.66,2.14) | 0.69 |
|  | Healthy-short | 1.00(0.80,1.26) | 1.00 | 1.04(0.82,1.32) | 0.85 |
|  | Healthy-long | 0.76(0.56,1.04) | 0.20 | 0.86(0.62,1.21) | 0.56 |
|  | Long-short | 1.13(0.64,2.03) | 0.77 | 1.37(0.74,2.53) | 0.50 |
|  | Long-healthy | 1.20(0.81,1.78) | 0.55 | 1.45(0.96,2.20) | 0.19 |
|  | Long-long | 0.77(0.45,1.34) | 0.55 | 0.95(0.54,1.70) | 0.92 |
| Arthritis-digestive class | Healthy-healthy | Ref | | Ref | |
|  | Short-short | 2.01(1.51,2.69) | <0.01 | 1.35(0.99,1.85) | 0.15 |
|  | Short-healthy | 1.07(0.71,1.60) | 0.82 | 0.86(0.56,1.31) | 0.62 |
|  | Short-long | 1.08(0.47,2.48) | 0.90 | 0.82(0.35,1.93) | 0.76 |
|  | Healthy-short | 1.74(1.27,2.36) | <0.01 | 1.52(1.10,2.08) | 0.04 |
|  | Healthy-long | 0.55(0.31,0.96) | 0.10 | 0.57(0.32,1.01) | 0.13 |
|  | Long-short | 0.56(0.17,1.86) | 0.54 | 0.57(0.17,1.94) | 0.56 |
|  | Long-healthy | 1.02(0.53,1.93) | 0.98 | 1.13(0.59,2.19) | 0.80 |
|  | Long-long | 1.18(0.56,2.47) | 0.77 | 1.23(0.57,2.63) | 0.72 |
| Respiratory class | Healthy-healthy | Ref | | Ref | |
|  | Short-short | 1.53(1.07,2.19) | 0.06 | 1.30(0.89,1.90) | 0.33 |
|  | Short-healthy | 1.05(0.65,1.69) | 0.89 | 0.95(0.58,1.54) | 0.89 |
|  | Short-long | 2.43(1.16,5.09) | 0.06 | 2.14(1.00,4.58) | 0.11 |
|  | Healthy-short | 1.62(1.12,2.34) | 0.03 | 1.50(1.03,2.18) | 0.08 |
|  | Healthy-long | 1.10(0.67,1.82) | 0.79 | 1.15(0.69,1.92) | 0.73 |
|  | Long-short | 1.98(0.84,4.65) | 0.25 | 2.03(0.85,4.83) | 0.23 |
|  | Long-healthy | 0.84(0.37,1.89) | 0.77 | 0.90(0.40,2.05) | 0.86 |
|  | Long-long | 1.24(0.54,2.85) | 0.74 | 1.39(0.60,3.24) | 0.64 |
| Multi-system morbidity class | Healthy-healthy | Ref | | Ref | |
|  | Short-short | 2.80(1.92,4.07) | <0.01 | 2.37(1.58,3.57) | <0.01 |
|  | Short-healthy | 2.05(1.29,3.27) | <0.01 | 1.90(1.16,3.11) | 0.04 |
|  | Short-long | 2.35(1.00,5.54) | 0.13 | 2.54(1.03,6.26) | 0.11 |
|  | Healthy-short | 2.65(1.80,3.92) | <0.01 | 2.56(1.70,3.85) | 3.92 |
|  | Healthy-long | 0.55(0.24,1.22) | 0.29 | 0.55(0.24,1.26) | 0.31 |
|  | Long-short | 3.23(1.41,7.40) | 0.02 | 3.58(1.45,8.83) | 0.02 |
|  | Long-healthy | 0.54(0.17,1.79) | 0.50 | 0.72(0.21,2.40) | 0.72 |
|  | Long-long | 0.56(0.13,2.36) | 0.59 | 0.64(0.15,2.77) | 0.69 |

Note: Model 1 adjusted for age and gender, Model 2 adjusted for age, gender, BMI, residence, education level, marital, smoke, drink, IADL, self-rated health, CESD-10, and napping time.

**Table S7 Association between prior changes in sleep duration and sleep trajectories with multimorbidity (additional adjustment for 2020 covariates).**

|  | OR(95%CI) | FDR P |
| --- | --- | --- |
| Changes in sleep duration |  |  |
| no change | Ref | |
| increased ≥1.5 h | 0.99(0.85,1.14) | 0.92 |
| increased ≥1.0 and <1.5 h | 1.02(0.88,1.18) | 0.83 |
| increased ≥0.5 and <1.0 h | 0.94(0.61,1.45) | 0.88 |
| decreased ≥0.5 and <1.0 h | 1.20(0.81,1.80) | 0.46 |
| decreased ≥1.0 and <1.5 h | 1.30(1.13,1.49) | <0.01 |
| decreased ≥1.5 h | 1.36(1.20,1.55) | <0.01 |
| Sleep trajectories |  |  |
| Healthy-healthy | Ref | |
| Short-short | 1.44(1.25,1.65) | <0.01 |
| Short-healthy | 0.99(0.85,1.16) | 0.96 |
| Short-long | 1.24(0.81,1.88) | 0.42 |
| Healthy-short | 1.44(1.27,1.63) | <0.01 |
| Healthy-long | 0.99(0.82,1.21) | 0.96 |
| Long-short | 1.37(0.97,1.95) | 0.14 |
| Long-healthy | 1.03(0.83,1.27) | 0.84 |
| Long-long | 0.94(0.68,1.31) | 0.83 |

Note: Model adjusted for age, gender, BMI, residence, education level, marital, smoke, drink, IADL, self-rated health, CESD-10, and napping time (residence, smoke and drink were collected in 2020).

**Table S8 Association of prior changes in sleep duration with multimorbidity patterns (additional adjustment for 2020 covariates).**

| Multimorbidity patterns  (ref:relatively healthy class ) | Changes in sleep duration | OR(95%CI) | FDR P |
| --- | --- | --- | --- |
| Metabolism class | no change | Ref | |
|  | increased ≥1.5 h | 0.97(0.81,1.15) | 0.81 |
|  | increased ≥1.0 and <1.5 h | 0.98(0.81,1.17) | 0.86 |
|  | increased ≥0.5 and <1.0 h | 1.23(0.74,2.05) | 0.52 |
|  | decreased ≥0.5 and <1.0 h | 1.31(0.82,2.08) | 0.35 |
|  | decreased ≥1.0 and <1.5 h | 1.25(1.06,1.47) | 0.01 |
|  | decreased ≥1.5 h | 1.29(1.11,1.50) | <0.01 |
| Arthritis-digestive class | no change | Ref | |
|  | increased ≥1.5 h | 0.79(0.63,0.99) | 0.09 |
|  | increased ≥1.0 and <1.5 h | 0.85(0.41,1.77) | 0.54 |
|  | increased ≥0.5 and <1.0 h | 0.65(0.19,2.22) | 0.77 |
|  | decreased ≥0.5 and <1.0 h | 1.01(0.54,1.89) | 0.98 |
|  | decreased ≥1.0 and <1.5 h | 1.29(1.04,1.59) | 0.04 |
|  | decreased ≥1.5 h | 1.18(0.97,1.44) | 0.17 |
| Respiratory class | no change | Ref | |
|  | increased ≥1.5 h | 1.07(0.79,1.44) | 0.75 |
|  | increased ≥1.0 and <1.5 h | 1.21(0.88,1.65) | 0.34 |
|  | increased ≥0.5 and <1.0 h | 0.43(0.10,1.79) | 0.36 |
|  | decreased ≥0.5 and <1.0 h | 0.90(0.35,2.31) | 0.86 |
|  | decreased ≥1.0 and <1.5 h | 1.20(0.89,1.62) | 0.34 |
|  | decreased ≥1.5 h | 1.43(1.09,1.87) | 0.02 |
| Multi-system morbidity class | no change | Ref | |
|  | increased ≥1.5 h | 0.88(0.63,1.22) | 0.57 |
|  | increased ≥1.0 and <1.5 h | 1.02(0.72,1.44) | 0.93 |
|  | increased ≥0.5 and <1.0 h | 1.82(0.80,4.16) | 0.23 |
|  | decreased ≥0.5 and <1.0 h | 1.95(0.91,4.19) | 0.15 |
|  | decreased ≥1.0 and <1.5 h | 1.43(1.05,1.95) | 0.05 |
|  | decreased ≥1.5 h | 1.70(1.28,2.26) | <0.01 |

Note: Model adjusted for age, gender, BMI, residence, education level, marital, smoke, drink, IADL, self-rated health, CESD-10, and napping time (residence, smoke and drink were collected in 2020).

**Table S9 Association of prior sleep trajectories with multimorbidity patterns (additional adjustment for 2020 covariates).**

| Multimorbidity patterns  (ref:relatively healthy class ) | Sleep trajectories | OR（95%CI） | FDR P |
| --- | --- | --- | --- |
| Metabolism class | Healthy-healthy | Ref | |
|  | Short-short | 1.10(0.94,1.29) | 0.37 |
|  | Short-healthy | 0.99(0.82,1.19) | 0.84 |
|  | Short-long | 1.06(0.66,1.70) | 0.82 |
|  | Healthy-short | 1.23(1.07,1.43) | 0.01 |
|  | Healthy-long | 0.85(0.67,1.08) | 0.31 |
|  | Long-short | 1.44(0.96,2.16) | 0.14 |
|  | Long-healthy | 1.03(0.79,1.33) | 0.92 |
|  | Long-long | 0.84(0.56,1.26) | 0.52 |
| Arthritis-digestive class | Healthy-healthy | Ref | |
|  | Short-short | 1.31(1.07,1.59) | 0.02 |
|  | Short-healthy | 0.82(0.63,1.07) | 0.23 |
|  | Short-long | 0.85(0.44,1.62) | 0.75 |
|  | Healthy-short | 1.45(1.20,1.75) | <0.01 |
|  | Healthy-long | 0.79(0.55,1.13) | 0.31 |
|  | Long-short | 1.25(0.72,2.18) | 0.55 |
|  | Long-healthy | 0.95(0.65,1.38) | 0.83 |
|  | Long-long | 1.32(0.81,2.14) | 0.38 |
| Respiratory class | Healthy-healthy | Ref | |
|  | Short-short | 1.45(1.11,1.90) | 0.02 |
|  | Short-healthy | 1.13(0.82,1.58) | 0.61 |
|  | Short-long | 1.52(0.77,3.00) | 0.31 |
|  | Healthy-short | 1.53(1.18,1.99) | <0.01 |
|  | Healthy-long | 1.38(0.94,2.01) | 0.15 |
|  | Long-short | 1.62(0.81,3.23) | 0.27 |
|  | Long-healthy | 0.93(0.55,1.56) | 0.82 |
|  | Long-long | 0.87(0.41,1.84) | 0.82 |
| Multi-system morbidity class | Healthy-healthy | Ref | |
|  | Short-short | 1.90(1.44,2.50) | <0.01 |
|  | Short-healthy | 1.17(0.82,1.67) | 0.54 |
|  | Short-long | 1.50(0.70,3.22) | 0.38 |
|  | Healthy-short | 2.18(1.67,2.85) | <0.01 |
|  | Healthy-long | 0.69(0.39,1.21) | 0.34 |
|  | Long-short | 2.81(1.49,5.33) | <0.01 |
|  | Long-healthy | 0.80(0.42,1.51) | 0.62 |
|  | Long-long | 0.43(0.13,1.41) | 0.28 |

Note: Model adjusted for age, gender, BMI, residence, education level, marital, smoke, drink, IADL, self-rated health, CESD-10, and napping time (residence, smoke and drink were collected in 2020).

**Table S10 Association between prior changes in sleep duration and sleep trajectories with multimorbidity (stratified by training and testing sets).**

|  | Training set | | Test set | |
| --- | --- | --- | --- | --- |
|  | OR(95%CI) | FDR P | OR(95%CI) | FDR P |
| Changes in sleep duration |  |  |  |  |
| no change | Ref | | Ref | |
| increased ≥1.5 h | 0.94(0.79,1.12) | 0.56 | 1.10(0.85,1.43) | 0.55 |
| increased ≥1.0 and <1.5 h | 0.97(0.81,1.16) | 0.78 | 1.15(0.88,1.50) | 0.48 |
| increased ≥0.5 and <1.0 h | 1.03(0.62,1.73) | 0.91 | 0.74(0.32,1.68) | 0.55 |
| decreased ≥0.5 and <1.0 h | 1.29(0.82,2.08) | 0.34 | 0.90(0.40,2.05) | 0.83 |
| decreased ≥1.0 and <1.5 h | 1.24(1.05,1.46) | 0.03 | 1.46(1.14,1.88) | <0.01 |
| decreased ≥1.5 h | 1.32(1.13,1.54) | <0.01 | 1.46(1.16,1.83) | <0.01 |
| Sleep trajectories |  |  |  |  |
| Healthy-healthy | Ref | | Ref | |
| Short-short | 1.42(1.21,1.67) | <0.01 | 1.48(1.15,1.90) | <0.01 |
| Short-healthy | 0.92(0.76,1.11) | 0.45 | 1.15(0.86,1.54) | 0.58 |
| Short-long | 1.49(0.91,2.52) | 0.18 | 0.82(0.39,1.78) | 0.77 |
| Healthy-short | 1.54(1.32,1.79) | <0.01 | 1.25(1.00,1.57) | 0.14 |
| Healthy-long | 1.11(0.88,1.41) | 0.45 | 0.78(0.55,1.11) | 0.39 |
| Long-short | 1.57(1.03,2.43) | 0.08 | 1.02(0.55,1.93) | 0.94 |
| Long-healthy | 0.99(0.76,1.28) | 0.93 | 1.07(0.73,1.56) | 0.87 |
| Long-long | 0.97(0.65,1.45) | 0.93 | 0.95(0.55,1.65) | 0.88 |

Note: Model adjusted for age, gender, BMI, residence, education level, marital, smoke, drink, IADL, self-rated health, CESD-10, and napping time.

**Table S11 Association of prior changes in sleep duration with multimorbidity patterns (stratified by training and testing sets).**

| Multimorbidity patterns  (ref: relatively healthy class) | Changes in sleep duration | Training set | | Test set | |
| --- | --- | --- | --- | --- | --- |
|  |  | OR(95%CI) | FDR P | OR(95%CI) | FDR P |
| Metabolism class | no change | Ref | | Ref | |
|  | increased ≥1.5 h | 0.86(0.70,1.06) | 0.28 | 1.24(0.90,1.70) | 0.38 |
|  | increased ≥1.0 and <1.5 h | 0.96(0.77,1.19) | 0.81 | 0.99(0.70,1.38) | 0.96 |
|  | increased ≥0.5 and <1.0 h | 1.28(0.72,2.30) | 0.52 | 1.07(0.37,3.13) | 0.96 |
|  | decreased ≥0.5 and <1.0 h | 1.44(0.85,2.44) | 0.29 | 0.84(0.31,2.26) | 0.87 |
|  | decreased ≥1.0 and <1.5 h | 1.20(0.99,1.46) | 0.16 | 1.38(1.02,1.85) | 0.11 |
|  | decreased ≥1.5 h | 1.19(0.99,1.43) | 0.15 | 1.48(1.13,1.95) | 0.02 |
| Arthritis-digestive class | no change | Ref | | Ref | |
|  | increased ≥1.5 h | 0.83(0.63,1.09) | 0.29 | 0.68(0.43,1.06) | 0.24 |
|  | increased ≥1.0 and <1.5 h | 0.87(0.65,1.16) | 0.45 | 0.97(0.63,1.50) | 0.96 |
|  | increased ≥0.5 and <1.0 h | 0.60(0.23,1.58) | 0.43 | 1.78(0.56,5.73) | 0.58 |
|  | decreased ≥0.5 and <1.0 h | 1.18(0.59,2.37) | 0.75 | 0.53(0.12,2.39) | 0.68 |
|  | decreased ≥1.0 and <1.5 h | 1.27(0.98,1.63) | 0.16 | 1.32(0.90,1.94) | 0.34 |
|  | decreased ≥1.5 h | 1.15(0.91,1.46) | 0.36 | 1.26(0.88,1.81) | 0.44 |
| Respiratory class | no change | Ref | | Ref | |
|  | increased ≥1.5 h | 1.05(0.73,1.50) | 0.84 | 1.16(0.67,2.00) | 0.82 |
|  | increased ≥1.0 and <1.5 h | 1.23(0.85,1.79) | 0.41 | 1.13(0.63,2.00) | 0.86 |
|  | increased ≥0.5 and <1.0 h | 0.58(0.14,2.48) | 0.59 | 0.00(0.00,0.00) | <0.01 |
|  | decreased ≥0.5 and <1.0 h | 1.30(0.50,3.43) | 0.71 | 0.00(0.00,0.00) | <0.01 |
|  | decreased ≥1.0 and <1.5 h | 1.22(0.85,1.75) | 0.41 | 1.19(0.69,2.06) | 0.79 |
|  | decreased ≥1.5 h | 1.38(1.00,1.91) | 0.14 | 1.54(0.95,2.50) | 0.22 |
| Multi-system morbidity class | no change | Ref | | Ref | |
|  | increased ≥1.5 h | 0.88(0.58,1.33) | 0.68 | 0.92(0.52,1.62) | 0.89 |
|  | increased ≥1.0 and <1.5 h | 1.13(0.74,1.73) | 0.71 | 0.79(0.43,1.47) | 0.71 |
|  | increased ≥0.5 and <1.0 h | 2.03(0.78,5.26) | 0.27 | 1.38(0.26,7.48) | 0.86 |
|  | decreased ≥0.5 and <1.0 h | 2.77(1.20,6.39) | 0.05 | 0.53(0.06,4.39) | 0.81 |
|  | decreased ≥1.0 and <1.5 h | 1.62(1.11,2.37) | 0.39 | 1.11(0.65,1.92) | 0.86 |
|  | decreased ≥1.5 h | 1.94(1.37,2.74) | <0.01 | 1.29(0.79,2.11) | 0.56 |

Note: Model adjusted for age, gender, BMI, residence, education level, marital, smoke, drink, IADL, self-rated health, CESD-10, and napping time.

**Table S12 Association of prior sleep trajectories with multimorbidity patterns (stratified by training and testing sets).**

| Multimorbidity patterns  (ref: relatively healthy class) | Sleep trajectories | Training set | | Test set | |
| --- | --- | --- | --- | --- | --- |
|  |  | OR（95%CI） | FDR P | OR（95%CI） | FDR P |
| Metabolism class | Healthy-healthy | Ref | | Ref | |
|  | Short-short | 1.11(0.92,1.17) | 0.42 | 1.05(0.78,1.40) | 0.90 |
|  | Short-healthy | 0.93(0.74,2.04) | 0.70 | 1.05(0.75,1.46) | 0.90 |
|  | Short-long | 1.18(0.68,1.53) | 0.70 | 0.82(0.31,2.21) | 0.88 |
|  | Healthy-short | 1.28(1.07,1.16) | 0.02 | 1.13(0.87,1.46) | 0.65 |
|  | Healthy-long | 0.87(0.65,2.55) | 0.50 | 0.80(0.51,1.25) | 0.62 |
|  | Long-short | 1.58(0.98,1.41) | 0.14 | 1.14(0.51,2.54) | 0.90 |
|  | Long-healthy | 1.03(0.75,1.59) | 0.92 | 0.94(0.59,1.49) | 0.90 |
|  | Long-long | 0.96(0.58,1.47) | 0.92 | 0.64(0.31,1.31) | 0.48 |
| Arthritis-digestive class | Healthy-healthy | Ref | | Ref | |
|  | Short-short | 1.25(0.98,1.14) | 0.16 | 1.49(1.03,2.14) | 0.12 |
|  | Short-healthy | 0.84(0.62,1.95) | 0.42 | 0.73(0.43,1.22) | 0.48 |
|  | Short-long | 0.94(0.46,1.85) | 0.92 | 0.61(0.14,2.74) | 0.74 |
|  | Healthy-short | 1.48(1.18,1.15) | <0.01 | 1.45(1.01,2.07) | 0.14 |
|  | Healthy-long | 0.74(0.48,2.36) | 0.32 | 0.94(0.49,1.77) | 0.92 |
|  | Long-short | 1.22(0.63,1.18) | 0.70 | 1.34(0.49,3.69) | 0.78 |
|  | Long-healthy | 0.71(0.43,2.22) | 0.32 | 1.48(0.83,2.63) | 0.42 |
|  | Long-long | 1.18(0.63,1.89) | 0.73 | 1.67(0.77,3.64) | 0.44 |
| Respiratory class | Healthy-healthy | Ref | | Ref | |
|  | Short-short | 1.63(1.18,1.94) | 0.01 | 1.10(0.67,1.80) | 0.88 |
|  | Short-healthy | 1.32(0.90,3.86) | 0.29 | 0.75(0.39,1.44) | 0.66 |
|  | Short-long | 1.73(0.78,2.36) | 0.32 | 1.31(0.35,4.87) | 0.88 |
|  | Healthy-short | 1.73(1.26,2.21) | <0.01 | 1.19(0.74,1.93) | 0.69 |
|  | Healthy-long | 1.39(0.88,3.60) | 0.29 | 1.42(0.73,2.78) | 0.59 |
|  | Long-short | 1.49(0.62,1.83) | 0.53 | 1.86(0.60,5.74) | 0.57 |
|  | Long-healthy | 0.96(0.50,2.56) | 0.92 | 0.89(0.36,2.16) | 0.90 |
|  | Long-long | 1.07(0.45,2.03) | 0.92 | 0.58(0.13,2.50) | 0.69 |
| Multi-system morbidity class | Healthy-healthy | Ref | | Ref | |
|  | Short-short | 1.98(1.42,1.86) | <0.01 | 1.65(1.01,2.71) | 0.14 |
|  | Short-healthy | 1.21(0.78,3.60) | 0.55 | 1.03(0.54,1.96) | 0.97 |
|  | Short-long | 1.42(0.56,3.31) | 0.63 | 2.21(0.56,8.74) | 0.54 |
|  | Healthy-short | 2.41(1.75,1.56) | <0.01 | 1.79(1.10,2.89) | 0.07 |
|  | Healthy-long | 0.81(0.42,6.07) | 0.70 | 0.48(0.16,1.45) | 0.44 |
|  | Long-short | 2.76(1.26,1.96) | 0.03 | 3.06(1.02,9.17) | 0.14 |
|  | Long-healthy | 0.92(0.43,2.47) | 0.90 | 0.57(0.17,1.95) | 0.65 |
|  | Long-long | 0.74(0.22,2.49) | 0.75 | 0.00(0.00,0.00) | <0.01 |

Note: Model adjusted for age, gender, BMI, residence, education level, marital, smoke, drink, IADL, self-rated health, CESD-10, and napping time.
